# Screening of the duplication 24 pb of *ARX* gene in Moroccan patients with X-linked Intellectual Disability

**DOI:** 10.1186/s13104-021-05526-7

**Published:** 2021-03-23

**Authors:** Yousra Benmakhlouf, Renaud Touraine, Ines Harzallah, Zeineb Zian, Kaoutar Ben Makhlouf, Amina Barakat, Naima Ghailani Nourouti, Mohcine Bennani Mechita

**Affiliations:** 1 Biomedical Genomics and Oncogenetics Research Laboratory, Faculty of Sciences and Techniques of Tangier, University Abdelmalek Essaadi, P.B.:416, Tangier, Morocco; 2 Molecular Genetics Laboratory, CHU, Saint Etienne, France; 3 BOUDRA Fertility Center (BFC) for Assisted Reproduction, Fez, Morocco

**Keywords:** Duplication 24 pb, *ARX*, X-linked intellectual disability, Nonsyndromic ID, Morocco

## Abstract

**Objective:**

Intellectual Disability (ID) represents a neuropsychiatric disorder, which its etiopathogenesis remains insufficiently understood. Mutations in the Aristaless Related Homeobox gene (*ARX*) have been identified to cause syndromic and nonsyndromic (NS-ID). The most recurrent mutation of this gene is a duplication of 24pb, c.428-451dup. Epidemiological and genetic studies about ID in the Moroccan population remain very scarce, and none study is carried out on the *ARX* gene. This work aimed to study c.428–451dup (24 bp) mutation in the exon 2 of the *ARX* gene in 118 males’ Moroccan patients with milder NS-ID to evaluate if the gene screening is a good tool for identifying NS-ID.

**Results:**

Our mutational analysis did not show any dup(24pb) in our patients. This is because based on findings from previous studies that found *ARX* mutations in 70% of families with NS-ID, and in most cases, 1.5–6.1% of individuals with NS-ID have this duplication. Since 1/118 = 0.0084 (0.84%) is not much different from 1.5%, then it is reasonable that this could a sample size artifact. A complete screening of the entire *ARX* gene, including the five exons, should be fulfilled. Further investigations are required to confirm these results.

## Introduction

Intellectual Disability (ID) is a neurodevelopmental disorder that represents an important concern of public health around the world. Its prevalence is estimated at 3% [1]. The Aristaless Related homeoboX (*ARX*) is an important gene responsible for X-Linked Intellectual Disability (XLID) [2] that belongs to the paired (Prd) class homeoprotein [3, 4]. It is an ortholog to the Drosophila aristaless homeobox gene [4], which is located in the chromosome Xp22.13. This gene spans 12.5 kb of genomic DNA and is composed of five exons [5] encoding a protein of 562 amino acids. This protein contains four polyalanine (polyA) tracts: 3 are encoded in exon 2 and 1 in exon 4 [6–8], as well as it contains highly conserved octapeptide, homeobox, and C-terminal domains [4, 9].

*ARX* gene is coding for a transcription factor [10, 11] and is expressed in the embryonic brain, endocrine pancreas, testes, and probably other tissues as well as in the adult brain, heart, skeletal muscle, and liver [4, 12]. Moreover, it has an important role in neurodevelopment [2].

Mutations of the *ARX* gene are the most frequent mutations of X-linked ID (XLID), and they are responsible for a wide phenotypic spectrum including S-XLID and NS-XLID forms [12–15], as well as X-linked lissencephaly with abnormal genitalia (XLAG), hydranencephaly with abnormal genitalia (HYD-AG), X-linked infantile spasm (ISSX), X-linked myoclonic seizures, spasticity and ID (XMESID), Partington syndrome (PRTS), Ohtahara syndrome, Proud syndrome, and idiopathic infantile epileptic dyskinesia encephalopathy (IEDE) [8, 16–20].

It was reported that mutations, deletions/insertions, duplications, missense, and nonsense mutations, of the *ARX* gene affect males more than females [21–23]. More than 60% of *ARX* mutations expand the first or second polyalanine tract [24]. The most known polyalanine expansion mutations are [c.428–451dup (24 bp) and c.333–334ins (GCG)] [11], they represent 46% of all identified mutations and have a vast pleiotropy. Conversely, the c.428–451dup (24 bp) mutation is the most frequent (45%) in the exon 2 [25, 26].

As far as we know, no studies about the mutations of the *ARX* gene have been reported in the Moroccan population up to date. Therefore, the aim of the present work was to study for the first time the c.428–451dup (24 bp) mutation in the exon 2 of the *ARX* gene among Moroccan ID males in order to evaluate if the gene screening is a good tool for identifying NS-ID.

## Main text

### Patients and methods

#### Patients

This study includes 118 males with milder NS-ID that were referred to three ID centers in Fez city, “Attawassol center for mentally retarded”, “Mafatih Arrahma” and “Prince Moulay Abdellah foundation”, from October 2014 to July 2017, and during April and July 2019. The mean age was 15.5 ± 6.4 years (from 2 to 31 years), and the IQs were < 70. These patients belonged to the same socio-economic situation in Morocco. The patient’s data was anonymous and confidential (a coding system was put in place).

Tutors or guardians were informed about the aims of the study, and they all signed informed consent. This study was approved by the Ethical Committee protocols of “University Hospital Ethics Committee in the Faculty of Medicine and Pharmacy in Fez, Morocco” (CEHUF). We have excluded, from our series, the patients presenting trisomy 21 or autism in order to prevent the overstatement of the etiologic diagnosis level. Physical, cognitive, and behavioral data were assessed and collected for each affected individual.

#### DNA extraction

Blood samples of NS-ID patients were collected at the three participating centers by qualified nurses. A total of 5 mL of blood have been collected from each patient in EDTA tubes for DNA extraction. Genomic DNA was isolated from blood leucocytes by the kit Wizard® Genomic DNA Purification and stored at − 20 °C in the Biomedical Genomics and Oncogenetics Research Laboratory at the Faculty of Sciences and Techniques of Tangier, Morocco.

#### Mutations screening

Before starting the PCR amplification, the extracted DNA concentration was measured using the Nanodrop spectrophotometer. All patients were screened for the 24 pb duplication (c.428–451dup), and the screening was performed by fragment analysis of PCR product of *ARX* exon 2, using Agilent 2100 bioanalyzer. The PCR amplification was carried out in a volume of 20 μl including 10 µl buffer, 1 µl of each primer, 0.5 µl Taq polymerase, and 50 ηg of DNA sample (Table 1). This was performed in the Molecular Genetics Laboratory at University Hospital Center, Saint Etienne, France.

**Table 1:** The PCR components and concentrations

Reagents	Concentration	Volume
H_2_O		5.5 µl
Buffer fail safe J	2.5 U/ µl	10 µl
Primer F	10 µM	1 µl
Primer R	10 µM	1 µl
Taq FailSafe	2.5 U/ µl	0.5 µl
DNA	50 ng/ µl	2 µl

Oligonucleotide primers were designed and tested for selectivity, specificity and sensitivity of target detection. These primers were self-designed using the Amplify software:

5′–3′.

Forward Primer: CAAGGCGTCGAAGTCTGGTGGTGC.

Reverse Primer: AGGGCGCCCCGTTCTCGCGGTA.

The parameters of PCR reaction were as follows: 96 °C for 6 s, 36 cycles of 95 °C for 30 s, 60 °C for 30 s and 72 °C for 1 min. A final extension step at 72 °C for 10 min ended the protocol.

For each series, a known mutated DNA with the 24 bp duplication is run in parallel as a control. If there is an amplification and if the fragment has the expected size, the series is validated.

### Results

#### Epidemiologic and clinical parameters

The mean age of our patients was 15.5 ± 6.4 years (from 2 to 31 years). All patients of our series were diagnosed with mild NS-ID (55 < IQ < 70). The IQ was evaluated using Wechsler standardized tests and/or a questionnaire established by the receiving center. Patients born from consanguineous parents represented 32.2%. We described an increased rate (20.42%) of consanguinity (first degree). Except for a 2 years old child, all patients were followed up in ID specialized institutions at the age of 7 years (legal age of education) that insure integration and education.

Language difficulty was the most common anomalies observed in our series with 54.4% (Table 2).

**Table 2:** Frequency of behavioral features of 118 ID patients

Manifestations	Number	Frequency (%)
Language difficulty	64	54.4
Epileptic seizures	27	22.88
Concentration problems	24	20.33
Nervousness	21	17.6
Aggressiveness	16	13.6
Hyperactivity and agitation	12	10.4

#### Screening of the Duplication c.428–451 (24 pb)

**Fig. 1:**
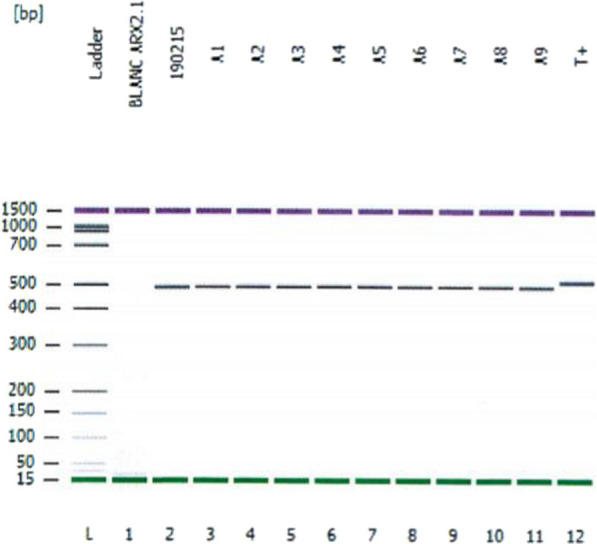
Electrophoresis of the PCR products for detection of the dup c.428–451 (24 pb) in exon 2 of the ARX gene in the affected male compared with the control (T+). A1-A6: males patients with XLID; Ladder: DNA 1000 Ladder; T+: Control (positive dup c.428–451 (24 pb). None c.428-451dup (24 pb) was found in our milder NS-ID patients

Screening of the most common mutation in exon 2, c.428–451 dup (24 bp), of the *ARX* gene was performed by PCR analysis. From 118 DNA tested, no mutation has been detected in our patients (Fig. 1).

### Discussion

Studies on ID are scarce in Arab countries, including Morocco [27], in particular studies focusing on the *ARX* gene. To the best of our knowledge, no studies about mutations in the *ARX* gene in the Moroccan population have been reported. The present work is the first to investigate the 24 bp duplication among patients with milder NS-ID in our country.

Indeed, X-linked ID may account for 10–12% of all ID cases. The *ARX* gene may be implicated in S-ID and NS-ID. *ARX* genetic mutations are known as one of the risk factors for various mental disorders, including NS-ID, due to its crucial role in the development of certain structures of the central nervous system (CNS) (cortex, hippocampus, dentate gyrus) [6, 13]. Thus, the *ARX* gene has been reported to be responsible for inherited and de novo mutations, including missenses and duplications/insertions [28]. The c.428-451dup24 mutation of the *ARX* gene had constituted the most frequent mutation, which predicted to cause an expansion of the polyalanine tract at amino acid position 144–155 from 12 to 20 alanine [13]. In this study, we have performed a screening of the c.428-451dup (24 pb) of the *ARX* gene in 118 males with NS-ID, and no mutation has been found in any patient of our series. Our results were similar to previous studies carried out in other populations from Belgium, Denmark, and Tunisia [6, 29]. The similarity observed between our findings and the Tunisian study might be explained by the shared North African origin and the same socioeconomic factors of both populations studied.

In contrast, a prior study conducted by Bienvenu et al. [13], to screen all coding exons of the *ARX* gene in 148 ID patients, revealed only one case with this recurrent mutation (24 pb duplication) in a sporadic case of ID. Another report demonstrated that only two dup24 were found in 1501 ID patients, and none was found in 151 families, which suggests that screening for this mutation in sporadic ID cases is very inefficient [28]. In addition, Gronskov et al. screened 682 samples from Denmark men aged 2–75 years and they identified only one recurrent mutation c.431–454dup (24 bp) among the XLID families [6]. Therefore, they suggested that *ARX* mutations were not a common cause of this disorder. This was in discordance with other earlier studies. Thus, literature data confirmed that screening of the mutation (c.428–451dup24) in exon 2 of the *ARX* gene must be performed in routine [30] since this mutation is responsible for 70% of NS-XLID families linked to Xp22.1 [13, 26, 30]. This mutation has been reported related to the inter-and intra-familial variabilities of expression and has been found in families with PRTS, ISSX, and XLIDS [5]. The estimated rate of its frequency is higher (6.1%) in XLID families [28].

Overall, our study showed that Moroccan patients with NS-ID did not have the *ARX* mutation (c.428–451dup24). Our findings supported those previously published studies suggesting that *ARX* screening should be performed not only for the exon 2 and the c.428-451dup24 mutation but also for all the five exons. Although our report showed that the polyalanine expansions in *ARX* are probably not a frequent cause of NS-XLID in our series, screening of all the mutations and all the exons is warranted to evaluate the prevalence of *ARX* mutations in our country.

The results of this study should be taken carefully due to some limitations. This study is limited by the monocentric nature since patients included were from centers located in only one city. Another limitation is the lack of some detailed clinical information of patients due to the socioeconomic factors, the high cost of analysis, which makes the diagnosis difficult and incomplete. According to the 2014 population census results, 12.5% of citizens in Morocco live under the national poverty line (the proportion is 7.9% in urban and 19.4% in rural areas) [31]. In addition, ID is generally unknown by Moroccan population which probably influenced the size of our series. Hence, we could not provide firm conclusions about if the *ARX* gene screening is a good tool for identifying NS-ID in our population. More investigations with extra samples from ID patients from multiple centers are required to validate these preliminary findings and to confirm them.

### Conclusion

Our study investigated, to the best of our knowledge, for the first time in Morocco, the mutation (c.428–451dup24) in exon 2 of the *ARX* gene and revealed no affected patient from our series. These findings highlight the need to establish a complete screening for the entire gene including the five exons. More studies should be done to confirm these results.

## Limitations

- Monocentric study: patients included were from three centers located in only one city.
- Lack of some detailed clinical information of patients due to the socioeconomic factors, the high cost of analysis, which makes the diagnosis difficult and incomplete.
- Small sample size: ID is generally unknown by the Moroccan population which probably influenced the size of our series.

## Data Availability

The datasets used and analyzed during the current study available from the corresponding author on reasonable request.

## Abbreviations

ARX: Aristaless related homeoboX
CEHUF: University Hospital Ethics Committee in the Faculty of Medicine and Pharmacy in Fez
CNS: Central nervous system
Dup: Duplication
HYD-AG: Hydranencephaly with abnormal genitalia
ID: Intellectual disability
IEDE: Idiopathic infantile epileptic dyskinesia encephalopathy
ISSX: X-linked infantile spasm
IQ: Intellectual quotient
NS-ID: Nonsyndromic intellectual disability
NS-XLID: Nonsyndromic X-linked intellectual disability
Prd: Paired
PRTS: Partington syndrome
S-XLID: Syndromic X-linked intellectual disability
XLAG-X: X-linked Lissencephaly with absent corpus callosum and ambiguous genitalia
